# Phylogeographical Analysis on *Squalidus argentatus* Recapitulates Historical Landscapes and Drainage Evolution on the Island of Taiwan and Mainland China

**DOI:** 10.3390/ijms13021405

**Published:** 2012-01-30

**Authors:** Jin-Quan Yang, Wen-Qiao Tang, Te-Yu Liao, Yang Sun, Zhuo-Cheng Zhou, Chiao-Chuan Han, Dong Liu, Hung-Du Lin

**Affiliations:** 1 Laboratory of Fishes, Shanghai Ocean University, Shanghai 201306, China; E-Mails: jqyang@shou.edu.cn (J.-Q.Y.); wqtang@shou.edu.cn (W.-Q.T.); sunyang_zhuiyi@163.com (Y.S.); dliu@shou.edu.cn (D.L.); 2 Department of Ichthyology, American Museum of Natural History, Central Park West at 79th Street, NY 10024, USA; E-Mail: swp0117@gmail.com; 3 College of Animal Sciences, Zhejiang University, Hangzhou 310029, China; E-Mail: zzcfyf@hotmail.com; 4 National Museum of Marine Biology and Aquarium, Pingtung 900, Taiwan; E-Mail: hancc@nmmba.gov.tw; 5 Department of Physical Therapy, Shu Zen College of Medicine and Management, Kaohsiung 821, Taiwan

**Keywords:** Mainland China, MtDNA, phylogeography, *Squalidus argentatu*, Taiwan

## Abstract

Phylogeographical analyses on *Squalidus argentatus* samples from thirteen localities within mainland China and Taiwan were conducted for biogeographic studies, as their dispersal strictly depends on geological evolution of the landmasses. A total of 95 haplotypes were genotyped for mtDNA cyt *b* gene in 160 specimens from nine river systems. Relatively high levels of haplotype diversity (*h* = 0.984) and low levels of nucleotide diversity (π = 0.020) were detected in *S. argentatus*. Two major phylogenetic haplotype groups, A and B, were revealed via phylogenetic analysis. The degree of intergroup divergence (3.96%) indicates that these groups diverged about 4.55 myr (million years) ago. Haplotype network and population analyses indicated significant genetic structure (*F_ST_* = 0.775), largely concordant with the geographical location of the populations. According to SAMOVA analysis, we divided these populations into four units: Yangtze-Pearl, Qiantang-Minjiang, Jiulong-Beijiang and Taiwan groups. Mismatch distribution analysis, neutrality tests and Bayesian skyline plots indicated a significant population expansion for lineage A and B, approximately dated 0.35 and 0.04 myr ago, respectively. We found strong geographical organization of the haplotype clades across different geographic scales that can be explained by episodes of dispersal and population expansion followed by population fragmentation and restricted gene flow.

## 1. Introduction

The Cyprinidae represent one of the most diverse freshwater fish groups and are a major component of the primary freshwater fish fauna of Africa, Eurasia and North America, comprising more than 220 genera and 2400 species [1]. Among the distribution of the Cyprinidae, China constitutes a vast area with remarkably high diversity, amounting to a total of more than 750 species [2]. According to the essential geohistorical events and ichthyofauna, Li [3] identified five major geographical districts in China, viz. (1) North, (2) West China, (3) Mongolia-Ninxia, (4) East China, and (5) South China Districts, with designations of subdivisions within each district. The Southern China District, including areas south of the Yangtze River and the South China Sea archipelagos, is well known for its highly diverse ecosystems and habitats, where the present distribution of freshwater species has been greatly influenced by past geological and hydrological events [3]. Many studies have explored the biogeography of southern China, and have described vicariant events that resulted in subsequent differentiation in salamanders [4], frogs [5], fishes [6], and birds [7]. However, among freshwater fishes, most of the works are centered in the Pearl and Yangtze Rivers. Studies dealing with freshwater taxa that are distributed in the east of the WuYi Mountains are scarce [6,8–12].

**Figure 1:**
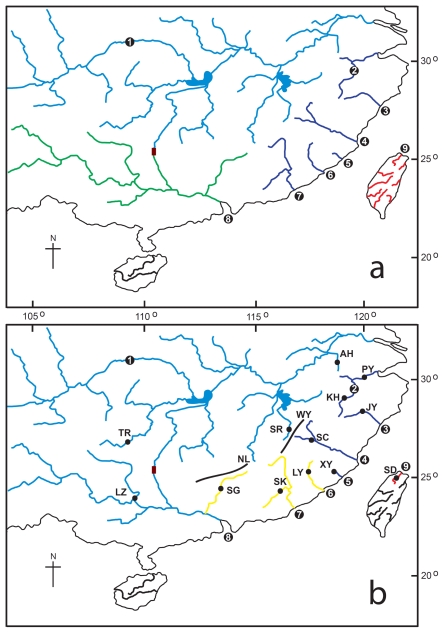
Maps showing (**a**) four sub-districts proposed by Li [3]. The Pearl River Subdistrict in green, ZheMin Sub-district in purple, Taiwan Sub-district in red, Kiang-Husi Sub-district in blue, and the Lingqu Canal connecting the Yangtze and Pearl Rivers in brown; 1 = Yangtze River, 2 = Qiantang River, 3 = Oujiang, 4 = Minjiang River, 5 = Mulan River, 6 = Jiulong River, 7 = Hanjiang, 8 = Pearl River, 9 = Tamsui River; (**b**) thirteen sampling sites of *Squalidus argentatus* and four units of samples based on SAMOVA analysis: Yangtze-Pearl group (in blue), Qiantang-Minjiang group (in purple), Jiulong-Beijiang group (in yellow), and Taiwan group (in red). See Table 1 for details of sampling sites. The bold lines indicate roughly the Wuyi (WY) and Nanling Mountains (NL).

The cyprinid genus *Squalidus* Sauvage and Dabry consists of a group of small-sized freshwater fishes broadly distributed in the lower and middle reaches of eastern Asia, including Korea, Japan, Vietnam, Hainan Island, Taiwan and most major drainages of mainland China [2]. Among all species of *Squalidus*, *S. argentatus* is the most extensively distributed both in China and Taiwan, where *S. argentatus* is restricted to the Tamsui River (Figure 1). Due to the broad distribution, *S. argentatus* is an ideal fish species to address the biogeography of this vast area in eastern Asia, especially to provide a better insight into the poorly studied eastern slope of the WuYi Mountains.

Taiwan is a continental shelf island (*sensu*) [13], which emerged because of the collision between the Philippine Sea and Eurasian continental plates approximately 5 myr (million years) ago (the Penglai Orogeny) [14,15]. During marine regression, freshwater fishes of the mainland dispersed to Taiwan via two paleo-river systems, one in northern Taiwan and the other in the south [16,17]. The paleo-river system, Guminjiang, in northern Taiwan is an extension of the Minjiang River with the Tamsui River of northern Taiwan as a southern branch [18]. *Squalidus argentatus* is one of the few cyprinid species distributed across the Taiwan Strait in addition to *Hemibarbus labeo*, *Sinibrama macrops*, *Pseudorasbora parva* and *Distoechodon tumirostris*. Population genetics regarding the trans-strait distribution of *H. labeo* and *S. macrops* have been addressed [12,19]. Unexpectedly, previous studies show that *H. labeo* and *S. macrops* of northern Taiwan are genetically associated with their conspecificities in the Qiantang River rather than those in the Minjiang River that connected to rivers of northern Taiwan during marine regression [12,19]. A close relationship of rivers of northern Taiwan to the Qiantang River may suggest a novel dispersal scenario other than the route of Guminjiang [16,17]. Additional studies of cyprinid species, e.g., *S. argentatus*, with similar trans-strait distribution could provide more substantial evidence to infer the dispersal pattern of freshwater fishes of Taiwan. In the present study, we collected *S. argentatus* of four geographic sub-districts based on Li’s hypothesis in our study: (1) Pearl River sub-district (South China District; populations SG and LZ); (2) ZheMin sub-district (South China District; populations KH, PY, JY, SC, XY, LY and SK); (3) Taiwan sub-district (South China District; population SD); and (4) Kiang-Husi Sub-district (East China District; populations AH, TR and SR) Figure 1 [3]. In order to reconstruct the complex dispersion pattern of southern China and Taiwan, phylogenetic analyses based on mitochondrial cyt *b* sequences of *S. argentatus* were performed. We thus elucidated the evolutionary relationships within *S. argentatus*, particularly focused on (1) describing the population genetic diversity; (2) discussing relationships between phylogenetic structures and geographic characters; (3) demonstrating the possible dispersal history between southern China and Taiwan; and (4) applying the interpretation of Management Units (MUs) [20,21] for the conservation of *S. argentatus*.

## 2. Results and Discussion

### 2.1. Results

#### 2.1.1. Genetic Diversity of *Squalidus argentatus* on the Island of Taiwan and Mainland China

Sequences of 1,140 bp of cyt *b* generated from 160 tissue samples of *Squalidus argentatus* were aligned and a total of 95 haplotypes were found. Alignment of all cyt *b* gene sequences revealed a total of 190 variable sites, among which 102 variable characters were singleton and 88 characters were parsimony-informative. Cyt *b* sequences were globally G deficient (15.0%), which is characteristic of the mitochondrial genome [22]; the other nucleotides had similar frequencies (A = 27.8%; C = 29.2%; T = 28.0%). This nucleotide composition has been frequently reported in cyt *b*-based studies on a variety of fishes, including cyprinids [8,23].

The sample size, number of haplotypes, values of nucleotide diversity (π), and haplotype diversity (*h*) within each population are presented in Table 1. Overall, the mean haplotype diversity (*h*) among the 160 samples was estimated to be 0.984, and the mean nucleotide diversity was 0.02063 (Table 1). Nucleotide diversity (π) among populations varied from 0.00105 (SD) to 0.00855 (SC); and haplotype diversity ranged from 0.628 (LY) to 1.000 (AH) Table 1. The mean haplotype diversity and nucleotide diversity are higher in mainland China (0.98, 0.0207) than in Taiwan (0.70, 0.0010).

#### 2.1.2. Phylogenetic Analysis

The existence of a phylogeographic structure was tested following Pons and Petit [24] by calculating two measures of genetic differentiation: *G*_ST_ and *N*_ST_. A comparison of the fixation indices *N*_ST_ and *G*_ST_ revealed a strong relationship between phylogeny and geography, with *N*_ST_ being much larger than *G*_ST_ (0.77874 and 0.12104, respectively). This result indicates the presence of phylogeographic structure, since the closely related haplotypes would be detected more frequently than those less closely related haplotypes in the same area [24].

**Figure 2:**
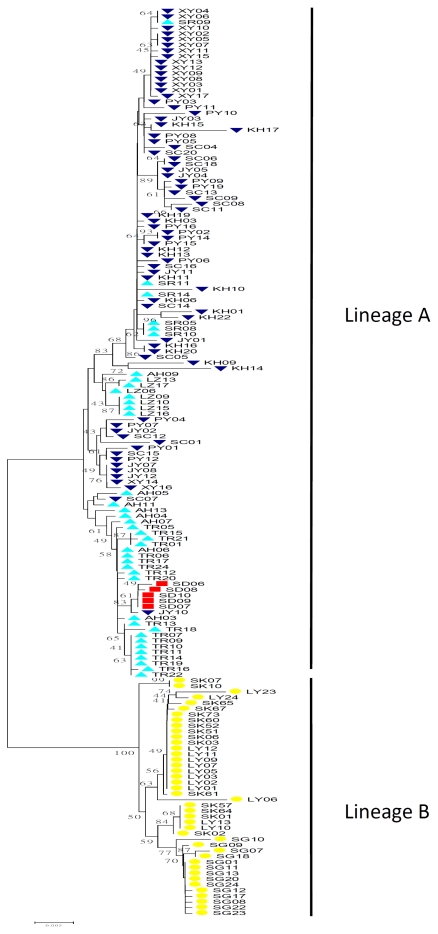
Neighbor-Joining tree of individual sequences of mtDNA cyt *b* gene in *Squalidus argentatus*. Numbers at the nodes indicate bootstrap values (expressed as percentage) with 1000 replicates. Thirteen sampling sites of *S. argentatus* and four units of samples based on SAMOVA analysis: Yangtze-Pearl group (triangle in blue), Qiantang-Minjiang group (inverted triangle in purple), Jiulong-Beijiang group (circle in yellow), and Taiwan group (square in red). Refer to Table 1 for the abbreviations of localities.

The NJ mtDNA gene tree indicated two strongly supported lineages (Lineages A and B; Figure 2), with Lineage A being widespread from the north of Minjiang River to the Yangtze River basin, as well as the Xijing River of the Pearl River system and the Tamsui River in Taiwan (populations AH, TR, SR, KH, PY, JY, SC, XY, LZ and SD). Lineage B consisted of individuals collected from the Jiulong River, Hanjiang River and the Beijiang River of the Pearl River system (populations LY, SK and SG). The lineage A comprised higher population genetic diversity (average, lineage A: π = 0.00993, *h* = 0.986; lineage B: π = 0.00534, *h* = 0.870).

#### 2.1.3. Population Genetic Analysis

Pairwise *F_ST_* tests indicated significant genetic differentiation among sampling locations (−0.007 to 0.953) after Bonferroni correction. Most of the pairwise *F_ST_* values were significant with a *P* value < 0.05 (Table 2). Differentiation was generally lower within lineages among the adjacent drainage systems. The overall standardized *F_ST_* value among all samples was 0.775. Between lineages, pairwise *F_ST_* values revealed highlighted significant values, varying from 0.829 to 0.953 (*P* < 0.01). According to four geographic districts, (1) Pearl River sub-district (populations SG and LZ); (2) ZheMin sub-district (populations KH, PY, JY, SC, XY, LY and SK); (3) Taiwan sub-district (population SD); and (4) Kiang-Husi Sub-district (populations AH, TR and SR), AMOVA analysis showed that most variances occurred among populations within geographic district (*P* < 0.001), contributing mainly to total variance (Table 3). The results of this study suggest that the four geographic districts of *S. argentatus* have not developed significant genetic structure so far. For the hierarchical analysis, populations were grouped according to their lineage assignment (Lineages A and B; *N =* 2). In the AMOVA, most of the molecular variance (75.74%) was attributable to variations between lineages, 11.40% attributable to variations among populations within groups, and 12.86% of the molecular variance related to variations within the same population. The SAMOVA revealed increasing *F_CT_* values when the numbers of groups increased (*K* = 2–6; *F_CT_* = 0.73–0.76). The best partitioning of the genetic diversity by SAMOVA was obtained when samples were divided into four units (*F_CT_* = 0.76, *p* = 0.000): (i) Yangtze-Pearl group: Yangtze River and Xijiang River (LZ) in the tributaries of the Pearl River (populations AH, TR, SR and LZ); (ii) Qiantang-Minjiang group: the river systems between Qiantang River and Minjiang River (populations KH, PY, JY, SC and XY); (iii) Jiulong-Beijiang group: three rivers in lineage B, including Jiulong River, Hanjiang River and Beijiang River (SG) in the tributaries of the Pearl River (populations LY, SK and SG); and (iv) Taiwan group: Taiwan Island (population SD) (Table 4). Reduced gene flow due to geographical structure was identified by comparing genetic distance and geographical distance among individuals by isolation using distance analysis. The Mantel test for *S. argentatus* indicated non-significant correlation between genetic and geographical distances (*r* = 0.1612, *P* = 0.000).

#### 2.1.4. Demographic History

**Figure 3:**
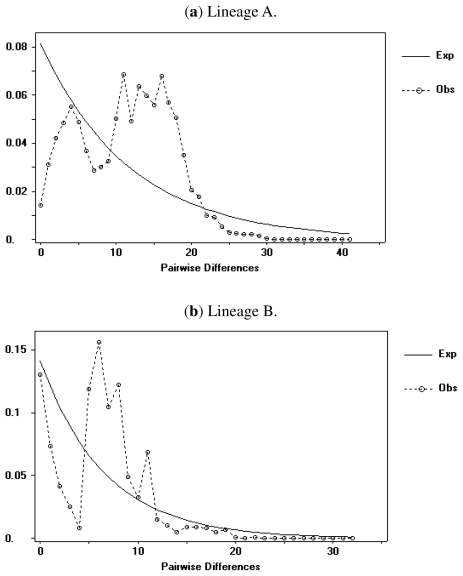
Mismatch-distribution analysis of *Squalidus argentatus* mtDNA haplotype sequences. A simulated Poisson distribution is indicated by a dotted line.

An examination of demographic histories revealed the marked differences between lineages and total populations under study. A signature of recent expansion was detected from total populations, as proved by the significant Fu’s test (*Fs* = −36.677, *P* < 0.000), although Tajima was not significant (*D* = −1.222, *P* > 0.10) (Table 1). Fu’s test has been shown to be much more sensitive in detecting population growth than Tajima’s test. The LAMARC analyses were roughly in agreement with tests for demographic expansion. Positive *g* values indicated that all populations were growing (Table 1). The model of population expansion could not be rejected because of its concordance with the expectation of historically expanding population when all samples were combined (SSD = 0.008, *P* = 0.72, for spatial expansion *vs.* SSD = 0.009, *P* = 0.84, for demographic expansion). This outcome was also supported by the low Harpending’s Raggedness index (*r* = 0.002, *P* = 0.74). Demographic analyses showed evidence of range expansions in two lineages. Tajima’s *D* and Fu’s tests were both significantly negative for two lineages, indicating that this species experienced a demographic expansion event under a neutral model. To characterize the expansion pattern further, a model of sudden demographic growth was fitted to the pairwise sequence mismatch distribution (Figure 3). In each lineage, there is a fit of the model of sudden expansion (parametric bootstrap goodness-of-fit tests did not reject the model).

**Figure 4:**
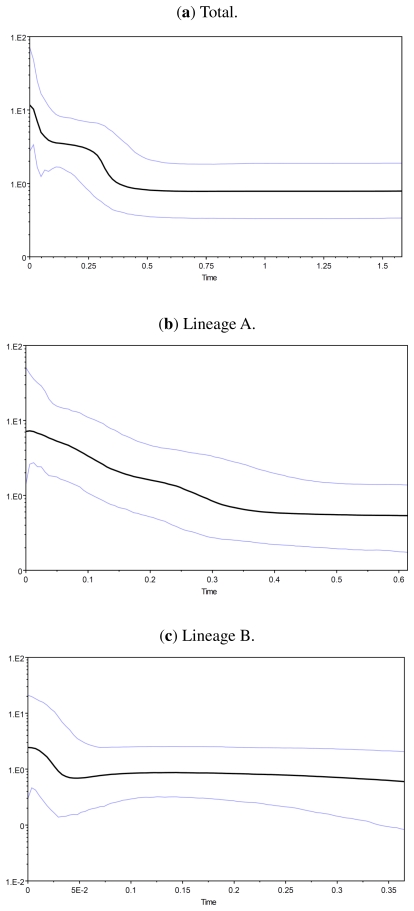
Bayesian skyline plot of the effective population sizes through time for *Squalidus argentatus*.

Bayesian skyline plots revealed a more complex demographic history. The effective sample size (ESS) for each of the Bayesian skyline analyses was greater than 200, suggesting that the 10 million generations were sufficient to determine the demographic history for *S. argentatus*. Skyline plots for *S. argentatus* indicate that recent population expansion began approximately 0.35 myr ago. Analyses of lineage A and lineage B samples both detected an initial population expansion starting at 0.35 and 0.04 myr ago, respectively. The lineage B exhibited a smaller effective population size compared to the lineage A, both before and after expansion (Figure 4).

#### 2.1.5. Molecular Dating

We used 1.05%/myr and a strict clock model implemented in BEAST to estimate the TMRCAs of different lineages and times of their separation [25]. The analyses with BEAST indicated that the time to TMRCA of all haplotypes dated back to 4.55 myr ago (ESS = 1146.175, 95% credibility interval 4.25–4.86 myr). Molecular dating estimated that the lineages A and B coalesced to the TMRCA 3.73 ± 0.24 myr and 1.98 ± 0.15 myr, respectively.

### 2.2. Discussion

#### 2.2.1. Genetic Variation within *Squalidus argentatus*

Nucleotide and haplotype diversities can provide information in the history of *Squalidus argentatus* populations. Grant and Bowen [26] interpreted four basic scenarios for population history based on values of haplotype and nucleotide diversity. Our results revealed high haplotype diversity and low to moderate nucleotide diversity in all populations (Table 1). This pattern of genetic diversity can be attributed to a recent population expansion after a low effective population size caused by founder events or bottlenecks [26]. Compared with previous studies of freshwater fishes in southern China, the genetic diversity of cyt *b* sequences is similar to that of the other species [6,8–11]. Genetic diversity is reduced in peripheral populations in comparison with central and more pristine populations, which may result from a small founder population or genetic drift or inbreeding depression [27]. At the lineage scale, the genetic diversity of the lineage A was higher than that of lineage B, implying that lineage A might represent the center of distribution. Furthermore, the genetic diversity in the Tamsui River in Taiwan was lower than that of the other populations in mainland China, suggesting a peripheral population. Considering that genetic diversity is usually directly related to population size [28], it is not surprising that the larger population size and deme size were found in mainland China. In contrast, the smaller population size found in Taiwan is in accordance with the short rivers, fast currents and fluctuating seasonal flows derived from the steep topography [29].

#### 2.2.2. Population Differentiation

Our results show that *S. argentatus* has a high level of genetic structuring, which is similar to many other widespread freshwater fishes observed in this region of southern China, such as *Zacco platypus* [8,10], *Opsariichthys bidens* [9–11], *Glyptothorax* [6] and *Hemibarbus labeo* [12]. Genetic structure and difference resulting from drainage isolation are easily detected in freshwater species because of their habitat isolation and limited dispersal capacity. When compared to Li’s hypothesis, a variance component analysis in AMOVA revealed that the molecular variability attributable to differences among Li’s geographical regions was comparatively low (Table 3). No significant differences were observed even when the grouping of geographical regions was rearranged based on Li’s hypothesis. Through homogeneous grouping, SAMOVA revealed four phylogeographical groups, (i) Yangtze-Pearl group (populations AH, TR, SR and LZ); (ii) Qiantang-Minjiang group (populations KH, PY, JY, SC and XY); (iii) Jiulong-Beijiang group (populations LY, SK and SG); and (iv) Taiwan group (population SD). This result indicated a high level of genetic structure, namely 76% of the total genetic diversity due to differences among populations (*F_ST_* = 0.775). Populations from different regions are characterized by a unique haplotype, which reflects the effects of geographic isolation [20]. The large sequence divergences between groups indicate that they have been isolated from one another for a considerable amount of time. We suggest vicariance following drainage isolation as an important mechanism for producing the differentiation among the groups.

#### 2.2.3. Phylogeography of *Squalidus argentatus*

The existence of two monophyletic groups of *S. argentatus* in South China is supported by pairwise genetic distances between groups (3.96%). Owing to the discrete geographical patterns, it can be inferred that the population genetic structure of *S. argentatus* in South China may have been significantly influenced by geological processes in the past. Allopatric populations will occupy recognizable, deeply-separated branches with time in an intraspecific gene tree. The amount of genetic differentiation may reflect mutations accumulated after the separation of populations and/or effects of lineage sorting from a polymorphic ancestral gene pool. Overall, lineage A and B could be associated with some degree of geographic structuring according to their distribution in South China. However, the Nanling and WuYi Mountains would have probably played a key role in geographically isolating representatives of lineage A and B, as barriers blocking dispersals between populations on either slope. The Nanling Mountains are the watershed of the Yangtze and Pearl River systems [29]. The *S. argentatus* populations distributed in Xijiang (LZ) are grouped into lineage A, and the population in Beijiang (SG) grouped into lineage B. However, Xijiang and Beijiang are the tributaries of the Pearl River system. The Xijiang population is more closely related to populations in the Yangtze and Minjiang Rivers than to the Beijiang population in terms of genetic structure. Close inter-relationships between populations from the Xijiang and Yangtze River are a common pattern observed in several cyprinids distributed in neighboring tributaries of these two river systems [11,30]. This peculiar trans-river affinity between the Xijiang and Yangtze Rivers might be a consequence of the Lingqu Canal, built more than 2200 years ago [31], connecting the Xiang River (Yangtze drainage) and the Xijiang River (Pearl River drainage). This trans-river affinity is also observed in *Zacco platypus* and *Opsariichthys bidens* [10].

Evolution and dispersal of freshwater fishes are closely related to paleogeography and especially to the history of basin connections, as a consequence of the geological development of landscapes. According to SAMOVA results, we divided the populations of the present study in mainland China into three groups, including the Yangtze-Pearl, Qiantang-Minjiang and Jiulong-Beijiang groups. The mountainous region, known as the WuYi Mountains, may play a critical role in blocking possible dispersal between drainages on both slopes. This is well supported by the phylogenetic and population genetic analyses that divide the western-slope drainage, the Yangtze River (Yangtze-Pearl group), and the eastern-slope drainage, the Minjing River (Qiantang-Minjiang group), into two separated clades (Figure 2).

The population collected from the Minjiang and Mulan Rivers (belonging to the lineage A) presented a genetic composition that was completely different from that of the Jiulong River (belonging to the lineage B), despite the short distance. This phylogenetic pattern is also observed in cyprinid fishes among which the Minjing and Jiulong Rivers serve as distribution boundaries. The Jiulong River is the northernmost border of *Hemibarbus medius*, *Puntius semifasciolatus* and *Sinibrama melrosei*, while the Minjiang River is the southernmost boundary of *Sinibrama macrops* and *Sarcocheilichthys parvus* [2]. Geological evidence indicates that most rivers in the southeast coastal districts, including the Jiulong and Hanjiang Rivers, were not present until the Quaternary [32,33]. This implies that the Jiulong and Hanjiang River are relatively young and fish species colonized these rivers from another source instead of the Minjing River.

The origin of the freshwater fish fauna of Taiwan, based on faunal similarity, suggested two (north and south) routes which connect Taiwan and mainland China, assuming the dispersal of freshwater species via land bridges during marine regression [16,17,34–36]. The phylogeography of the cyprinid fish distributed in rivers of western Taiwan (*Varicorhinus barbatulus*, [34]; *Formosania lacustre*, [35]; *Cobitis sinensis*, [23]) indicates that the Miaoli Plateau was the last region isolated from the Minjiang River in mainland China. However, freshwater fishes of the Tamsui River may have another dispersal route. The haplotypes derived from individuals of the Tamsui River are monophyletic and nested with those derived from mainland China, and a haplotype of Qiantang River is at the base of the clade (Figure 2). This result might indicate that the Qiantang River is a source population for colonization of *S. argentatus* in Taiwan. *Squalidus argentatus*, *H. labeo* and *S. macrops* in Taiwan are all only distributed in the Tamsui River. Our phylogeographic pattern of *S. argentatus* coincides with *H. labeo* and *S. macrops*, with a sister group in the Qiantang River instead of the Minjiang River [12,19]. Since *S. argentatus* is limited to the Tamsui River in northern Taiwan and has failed to spread further, dispersal from mainland China is hypothesized to be a recent event.

The BEAST analysis estimated an approximate age of 4.55 ± 1.0 myr for all haplotypes in South China, a time close to the divergence of *O. bidens* populations in the same region. Concordant results for the geographical divergence support a common history for these co-distributed freshwater taxa [11]. Geological evidence indicates that the formation of the Nanling Mountains have emerged since 11.06–8.04 myr ago (Ministry of Geology and Mineral Resources “Nanling Project” Special Group 1988). Concordant phylogeographic patterns and similar population age estimations suggest that similar events have shaped the distributions and genetic population structures of several freshwater fishes in southern China (e.g., *G. fokiensis*; [6]; *O. bidens*; [11]; *H. labeo*; [12]).

#### 2.2.4. Demographic History

In our analysis, both the neutrality tests and the mismatch distribution analysis, indicated population expansion in both lineage A and B. In fact, Tajima’s D and Fu’s Fs tests significantly rejected the selective-neutrality hypothesis, as well as smooth unimodal mismatch distributions for two lineages. This is in agreement with the observation reported for cyt *b* in other freshwater fishes in southern China [6,11,12]. The historical demographic analysis carried out in this study indicated that the effects of Pleistocene climatic changes on the population dynamics of *S. argentatus* were lineage-specific and depended predominantly on the colonization history and geography of the two evolutionarily independent clades. Furthermore, the Bayesian skyline plots indicated that the population size increased, particularly around 0.30 myr ago in lineage A. Estimation of population expansion time for lineage A is much older than that of lineage B. Demographic expansions into these river systems in lineage B may have occurred more recently, and this is concordant with the molecular dating from mtDNA markers. This expansion started approximately 0.05 myr ago in the late Pleistocene period in lineage B.

#### 2.2.5. Implications for Conservation

According to the model proposed by Moritz [21], evolutionary significant units (ESUs) are designated on the basis of reciprocal monophyly at mitochondrial markers, while management units (MUs) are identified by significant differences in allele frequency distributions and significant divergence in mitochondrial or nuclear loci. Phylogeography and genetic diversity data can contribute to the development of effective conservation strategies. The importance of fish diversity in South China has been largely recognized but very few efforts have been made to establish a basis for their conservation. The two reciprocally monophyletic lineages of *S. argentatus*, support the idea that future conservation plans should be aimed at managing the populations of both lineages independently and, furthermore, be considered as two ESUs. For the different MUs, we suggest that protection is required synchronously because of their genetic uniqueness. We suggest that four regions identified by SAMOVA should be regarded as different MUs, conforming to the major zoological regions of mainland China and Taiwan.

## 3. Experimental Section

### 3.1. Sampling

From 2008 to 2010, 160 individuals of *Squalidus argentatus* were collected from 13 populations in 9 drainages, including 12 populations in Mainland China and one population in Taiwan (Table 1; Figure 1). Total genomic DNA was isolated from muscle tissue or fins preserved in 95% ethanol, by proteinase K digestion at 37 °C or 55 °C. DNA was purified by standard phenol: chloroform extraction and ethanol precipitation [37].

### 3.2. Molecular Analyses

The entire cytochrome *b* gene was amplified using a polymerase chain reaction (PCR) with primers SRCD1 (5′-CTCGGATTTTAACCGAGACC-3′) and H15915 (5′-CTCCGATCTCCGGATTACAAGAC-3′) [38]. Each 100 μL PCR reaction contained 10 ng template DNA, 10 μL 10× reaction buffer, 10 μL dNTP mix (8 mM), 10 pmol of each primer, and 4U of *Taq* polymerase (Promega, Madison, WI, USA). Reactions of PCR amplification were conducted in a thermal cycler (Eppendorf Mastercycler) using the following conditions: one cycle of denaturation at 95 °C for 4 min, 30 cycles of denaturation at 94 °C for 45 s, annealing at 48 °C for 1 min 15 s, and extension at 72 °C for 1 min 30 s, followed by 72 °C extension for 10 min and 4 °C for storage. PCR products were purified by electrophoresis in a 1.0% agarose gel using 1X TAE buffer. The gel was stained with ethidium bromide and the desired DNA band was cut and eluted using the Agarose Gel Purification Kit (QIAGEN, Valencia, CA, USA). Products of the cycle sequencing reactions were conducted by Sangon Biotec (Shanghai) Co., Ltd. with an ABI PRISM 3730 sequencer using the BigDye Terminator kit (Applied Biosystems). The primers used for sequencing were the same as those for PCR amplification. All sequences have been deposited in GenBank under the following inclusive accession numbers: JQ421136-JQ421295.

### 3.3. Data Analyses

#### 3.3.1. Genetic Diversity, Phylogenetic and Phylogeographic Analysis

The entire cyt *b* gene of mtDNA sequences obtained were aligned with the program CLUSTAL X 1.81 [39] and then optimized manually. Genetic diversity was measured for all samples and for each basin grouping using haplotype diversity (*h*) [40], nucleotide diversity (θ) [41] and nucleotide diversity (π) [42] using the software DnaSP 5.0 [43].

The sequence data was analyzed with the Neighbour-joining (NJ) method using Kimura 2-parameter distance method with MEGA 4 [44]. Neighbour-joining tree nodes and branch lengths were statistically tested using a bootstrap method of 1000 replicates approach, and an interior branch test, respectively. Bayesian analysis was carried out using MrBayes, version 3.1.2 [45]. Best-fit models for the Bayesian analysis were inferred by hierarchical likelihood ratio tests using MRMODELTEST, version 2.3 [46]. Markov chain Monte Carlo simulations were run for 5,000,000 generations with trees sampled every 1000 generations. Then, Bayesian posterior probabilities were estimated after omitting the initial 1,000,000 generations.

The estimates of geographic spread were compared with those obtained by the phylogeographic differentiation test of Pons and Petit [24]. As an overall assessment of geographical structure affecting the population differentiation, a comparison of the two fixation indices, *G*_ST_ and *N*_ST_ was carried out using DnaSP 5.0 [43].

#### 3.3.2. Historical Demography

To infer population demographic history of *S. argentatus* several methods were used including, Tajima’s *D* statistics [47], Ramos-Onsins and Rozas’ *R*_2_ [48] and Fu’s *FS*-test [49] of neutrality. The frequency distribution of pairwise differences between mtDNA haplotypes (*i.e*., mismatch distribution), estimated exponential growth rate (g) with program LAMARC [50], and Bayesian skyline plots (BSP) were evaluated [51]. Departures from neutrality of *R*_2_, Fu’s *FS* and Tajima’s *D* test indicate recent population expansions under assumptions of neutrality [47–49]. Significance of *R*_2_, Fu’s *FS* and Tajima’s *D* values was evaluated using the coalescent algorithm implemented in DnaSP 5.0 [43], comparing the observed value with a null distribution generated by 10,000 replicates, and giving an empirical population sample size and the observed number of segregating sites. The demographic history of *S. argentatus* was explored using mismatch analysis [52] of cyt *b* mitochondrial sequences. This method is based on the premise that compared with constant population size, population growth or decline leaves distinctive signatures in DNA sequences. If the cyt *b* locus examined here is neutral and has been transmitted under equilibrium conditions, then a multimodal distribution of haplotypes should result. Alternately a unimodal distribution (*i.e.*, a large number of closely related haplotypes) could indicate non-equilibrium conditions, especially population expansion. To compare observed distributions with those expected under the expansion model, we calculated the sum of square deviation (SSD) and the Harpending’s raggedness index [53]. In order to recover the demographic history, coalescence methods require that an initial demographic model be specified. We used the HKY + Γ + I model to construct Bayesian skyline plots in BEAST version 1.5.3 for each lineage [54]. This coalescent-based approach estimates the posterior distribution for effective population size at intervals along a phylogeny, thereby allowing inferences of population fluctuations over time. We ran 10^6^ generations. Burn-in and plots for each analysis were visualized using Tracer version 1.5 [55].

#### 3.3.3. Population Genetic Differentiation

Pairwise *F_ST_* values and analysis of molecular variance (AMOVA) were used to assess the population configuration and the geographical pattern of population subdivision, as implemented by Arlequin Version 3.5 [56]. Pairwise *F_ST_* values among sites were calculated and assessed for significance by comparison with 10,000 permutations of data. For the hierarchical analysis, populations were grouped according to the geographical districts. For the AMOVA hierarchical analysis, populations were grouped according to main lineages (*N* = 2) and four sub-districts (*N* = 4): (1) Pearl River sub-district (populations SG and LZ); (2) ZheMin sub-district (populations KH, PY, JY, SC, XY, LY and SK); (3) Taiwan sub-district (population SD); and (4) Kiang-Husi Sub-district (populations AH, TR and SR). Statistical significance of differentiation at the three levels was quantified and tested using Arlequin Version 3.5 [56]. The program SAMOVA [57] was also used to identify groups of adjacent sampling sites with maximum extent of genetic differentiation, as summarized by the *F_CT_* statistic. This analysis also delineates genetic barriers between the inferred groups. We performed these analyses based on 500 simulated annealing steps, and compared maximum indicators of differentiation (*F_CT_* values) when the program was instructed to identify *K* = 2 through *K* = 6 partitions of the sampling area for each sampling area within each analysis. Mantel tests [58], implemented in the program AIS [59] with 1000 permutations, were performed to examine the correlations between geographical and genetic distance.

#### 3.3.4. Molecular Dating

A Bayesian analysis of combined data was also used to estimate the divergence times of the major lineages to the most recent common ancestor (TMRCA) by software BEAST version 1.5.3 [54]. Based on multiple fossil data and sequence comparisons [25], a divergence rate of about 1.05 % per million years was estimated for the cyt *b* gene of cyprinids and hence to obtain absolute values of TMRCA. All the analyses were performed using the HKY model of nucleotide substitution. Adequate sampling and convergence to the stationary distribution was checked using TRACER Version 1.5 [55]. Posterior estimates of parameters were all found to be distinctly unimodal (although with wide 95% highest posterior densities), and all parameters appeared to be identifiable, despite the relatively low information content in the sequences and the small age range of the sequences.

## 4. Conclusions

Our study showed that *S. argentatus* has a high level of genetic structure among geographical populations. A strong phylogeographic structure due to restricted gene flow among geographic populations was identified in this species, suggesting that the WuYi and Nanling Mountains have formed barriers for this species. We divided all populations of *S. argentatus* into four groups, consisting of Yangtze-Pearl group, Qiantang-Min group, Jiulong-Beijiang group and Taiwan group. According to the network, there are at least two major migratory routes in mainland China, one from the Yangtze-Pearl group to the Jiulong-Beijiang group and the other to the Qiantang-Min group. The population in Taiwan came from the Qiantang River in mainland China. Demographic analyses implied a population expansion occurred during the recent history of the species. Conforming to the major phylogeographical regions of mainland China and Taiwan, four Management Units have been identified and should be considered for future management projects.

## Figures and Tables

**Table 1:** Summary of sample size, number of haplotypes, haplotype diversities (*h*), nucleotide diversity (π, θ), Tajima’s *D* and Fu’s (*Fs*) test, Ramos-Onsins and Rozas’ *R*_2_ and exponential growth rate (*g*) for mt DNA Cyt *b* region sequences in each population.

Region subregion	Populations (Abbreviation)	Sample size	Haplotype numbers	Haplotype Diversity (*h*)	Nucleotide diversity (π)	Nucleotide diversity (θ)	Tajima’s D*	Fu’s FS	*R*_2_	*g*
**Mainland China**		155	92	0.983	0.02073	0.03295	−1.19838	−33.072***	0.0584	
**Yangtze River**		33	20	0.939	0.00659	0.00908	−0.99662	−5.021**	0.0584	
	AnHua (AH) I	8	8	1.000	0.00583	0.00677	−0.72263	−3.129*	0.1136	1259.96
	Tongren (TR) I	19	10	0.854	0.00214	0.00301	−1.04808	−3.859*	0.0913	2180.86
	ShangRao (SR) I	6	3	0.733	0.00140	0.00154	−0.46983	0.615	0.2894	569.91
**Qiantang River**		31	25	0.981	0.00834	0.01998	−2.20960**	−10.765***	0.0584	
	Kaihua (KH) II	15	11	0.933	0.00836	0.01565	−2.01412*	−1.083	0.0766	273.56
	PingYao (PY) II	16	14	0.983	0.00787	0.01005	−0.90881	−4.471*	0.0998	666.16
**Oujiang River**										
	Jinyun (JY) II	10	7	0.911	0.00823	0.00806	0.09713	0.724	0.1648	217.45
**Minjiang River**										
	Shunchang (SC) II	15	14	0.990	0.00855	0.01052	−0.79692	−5.112**	0.1015	580.04
**Mulan River**										
	Xianyou (XY) II	17	9	0.860	0.00337	0.00493	−1.24407	−1.450	0.0959	31.26
**Jiulong River**										
	Longyan (LY) III	13	5	0.628	0.00490	0.00820	−1.75948	2.843	0.1268	90.72
**Hanjiang River**										
	Jiaoling (SK) III	15	7	0.819	0.00459	0.00459	0.00760	0.878	0.1424	98.89
**Pearl River**										
Xijiang River	Shaoguan (SG) III	14	10	0.890	0.00222	0.00441	−2.04167*	−5.409**	0.0959	7760.08
Beijiang River	Liuzhou (LZ) I	7	4	0.714	0.00209	0.00179	0.82563	0.205	0.2209	504.63
**Taiwan**										
**Tamsui River**	Tamsui (SD) IV	5	3	0.700	0.00105	0.00126	−1.04849	−0.186	0.2667	9936.337
**Total**		160	95	0.984	0.02063	0.03323	−1.22243	−36.677***	0.0584	193.36

I Yangtze-Pearl group;

II Qiantang-Minjiang group;

III Jiulong-Beijiang group;

IV Taiwan group (SAMOVA);

* *P* < 0.05;

** *P* < 0.01;

*** *P* < 0.001;

*g* estimated with LAMARC.

**Table 2:** Matrix of pairwise *F_ST_* *(below diagonal) and P* values (above diagonal) between 13 populations based on mtDNA in *Squalidus argentatus*. Referred to Table 1 for the abbreviations of localities.

	AH	TR	SR	KH	PY	JY	SC	XY	LY	SK	SG	LZ	SD
**AH**		0.000	0.000	0.000	0.000	0.000	0.000	0.000	0.000	0.000	0.000	0.000	0.000
**TR**	0.300		0.000	0.000	0.000	0.000	0.000	0.000	0.000	0.000	0.000	0.000	0.000
**SR**	0.671	0.858		0.459	0.126	0.009	0.036	0.036	0.000	0.000	0.000	0.000	0.000
**KH**	0.486	0.693	−0.007		0.027	0.000	0.000	0.000	0.000	0.000	0.000	0.000	0.000
**PY**	0.427	0.660	0.053	0.052		0.162	0.333	0.000	0.000	0.000	0.000	0.000	0.000
**JY**	0.279	0.600	0.236	0.150	0.028		0.162	0.000	0.000	0.000	0.000	0.000	0.000
**SC**	0.410	0.648	0.098	0.085	−0.001	0.022		0.000	0.000	0.000	0.000	0.000	0.000
**XY**	0.661	0.811	0.251	0.213	0.194	0.319	0.246		0.000	0.000	0.000	0.000	0.000
**LY**	0.845	0.904	0.904	0.839	0.835	0.833	0.829	0.897		0.243	0.000	0.000	0.000
**SK**	0.848	0.903	0.905	0.841	0.837	0.836	0.831	0.897	0.008		0.000	0.000	0.000
**SG**	0.901	0.939	0.953	0.877	0.873	0.880	0.866	0.931	0.490	0.463		0.000	0.000
**LZ**	0.364	0.757	0.814	0.500	0.457	0.399	0.442	0.717	0.882	0.884	0.939		0.000
**SD**	0.441	0.603	0.919	0.651	0.629	0.558	0.611	0.826	0.893	0.893	0.949	0.841	

**Table 3:** AMOVA results for testing genetic subdivision between populations using mtDNA among four sub-districts. (1) Pearl River sub-district (populations SG and LZ); (2) ZheMin sub-district (populations KH, PY, JY, SC, XY, LY and SK); (3) Taiwan sub-district (population SD); and (4) Kiang-Husi Sub-district (populations AH, TR and SR).

Source of variation	Variance components	Percentage of variation
Among geographic district	1.03198	7.96
Among populations within geographic district	9.06970 ***	69.97
Within populations	2.86151 ***	22.07

* *P* < 0.05;

** *P* < 0.01;

*** *P* < 0.001.

**Table 4:** SAMOVA results for testing genetic subdivision between populations of using of mtDNA among four units. (i) Yangtze-Pearl group (populations AH, TR, SR and LZ); (ii) Qiantang-Min group (populations KH, PY, JY, SC and XY); (iii) Jiulong-Beijiang group (populations LY, SK and SG); and (iv) Taiwan group (population SD).

Source of variation	Variance components	Percentage of variation
Among groups	9.83421 *	77.40
Among populations within zoogeographic zone	0.01060 ***	0.08
Within populations	2.86151 ***	22.52

* *P* < 0.05;

** *P* < 0.01;

*** *P* < 0.001.
